# Correlation Between Oral Health Status and Oral Health Impact Profile in Children With Special Needs: A Cross-Sectional Study

**DOI:** 10.7759/cureus.93278

**Published:** 2025-09-26

**Authors:** Sakshi Aggarwal, Mansi Atri, Nithya S, Jitin Kharbanda, Minni Chadha, Anantdeep Singh, Meenu Mittal, Chaity Sarkar

**Affiliations:** 1 Public Health Dentistry, ESIC Dental College and Hospital Rohini, New Delhi, New Delhi, IND; 2 Oral Pathology and Microbiology, ESIC Dental College and Hospital Rohini, New Delhi, New Delhi, IND; 3 Periodontics, ESIC Dental College and Hospital Rohini, New Delhi, New Delhi, IND; 4 Paediatric Dentistry, ESIC Dental College and Hospital Rohini, New Delhi, New Delhi, IND

**Keywords:** disabilities, oral health awareness, oral health impact profile, oral health status, special needs children

## Abstract

Aim: The study aims to correlate the oral health status and oral health impact profile (OHIP) of children with special needs.

Objectives: The study's objectives are to assess and compare the oral health status and OHIP of children with special needs using the Oral Health Impact Profile - 14 items (OHIP-14) questionnaire and through clinical examination.

Material and methods: One hundred thirty-five children with special needs under 13 years of age attending a special school in Dwarka, New Delhi, India, were included in the study. They were surveyed with the help of a questionnaire and clinical examination. In addition to sociodemographic factors, the children were assessed for the type of disability. Their oral hygiene was assessed using questions on frequency of brushing, other dental aids, and the reason for dental visits. The oral health impact profile was assessed using the OHIP-14 questionnaire, and the total score was obtained by adding the individual scores of seven domains. Oral health was examined using the decayed, missing, and filled teeth (DMFT) index, the gingival index, and the plaque index. The total DMFT score was calculated by adding the scores of each tooth. Oral hygiene was recorded as excellent, good, fair, or poor as per plaque index scores. Gingival disease was recorded as mild, moderate, or severe as per the gingival index scores. OHIP scores were correlated with DMFT scores, gingival index, and plaque index scores to assess the oral health-related quality of life (OHRQoL) of these children.

Results: A total of 135 subjects studying in a special school in Dwarka were studied. Out of the total subjects, 104 (77%) were male and 31 (23%) female. The majority of the study population (113, 83.7%) were in the age group of 10-13 years, 17 (12.6%) were in seven to nine years, and five (3.7%) were in four to six years. Regarding basic oral hygiene, the majority (72.6%) of children (98) could do household chores on their own, 104 (77%) brushed only once, and 74 (54.8%) used mouthwash in addition to brushing. Out of all children, 106 (78.5%) had never visited a dentist. The study population included 69 (51.1%) autistic children. The mean OHIP score was 5.05 ± 6.28, and the mean DMFT score was 2.96 ± 2.29. On correlating OHIP scores with indices, statistical significance was seen in correlating DMFT with functional limitation score (p = 0.048), DMFT with handicap score (p = 0.219), and gingival score with handicap score (p = 0.230).

Conclusion: The study reveals that children with special needs have poor oral hygiene with increased risk of caries and gingival disease. However, there is no impact on their quality of life. The main reason is the acceptance of poor oral hygiene by both the children and their caregivers. Thus, the need of the hour is to sensitize the caregivers and parents of these children regarding the importance of oral health through various oral health programs.

## Introduction

Oral health is an essential part of overall health. The health of children largely depends on their oral hygiene and dental health, and poor dental health can impact their quality of life.

Children with special needs can be defined as those children who “have or are at increased risk for chronic developmental, physical, emotional, or behavioral conditions and who also require health and related services of an amount or a type beyond what is required by children normally” [1]. These children form an important part of the population. According to the Census 2011, 2.21% of the total population in India includes disabled people, estimated to be about 26.8 million. According to the report published by the National Statistical Office, Ministry of Statistics and Programme Implementation, Government of India, on March 31, 2021, 20% of disabled people reported disability in movement, 19% had impairment in vision and auditory problems, and 7% had speech impairment. The report states that 6% of the disabled population experiences “mental retardation” or intellectual disability, which makes it difficult for them to understand, comprehend, or communicate [2].

The special needs children have neglected oral health and are at greater risk for the development of oral disorders throughout their lives. Children with developmental, intellectual, physical, and mental disabilities who are not able to cooperate or understand preventive oral health measures are more vulnerable [3].

The children have a very slow learning pace, which makes it difficult for them to understand various methods of maintaining good oral hygiene, such as simple brushing techniques. Another major cause of poor oral health is denied dental treatment due to their uncooperative behavior and the limited interest of caregivers in their oral health, with more focus placed on their medical problems.

The oral health status of children with special needs largely depends on their living conditions, age, type of disability, and severity of impairment. Other factors causing poor oral health include poor or no knowledge of oral healthcare, the education level of parents and caregivers, and socioeconomic status among parents and caregivers [4].

Children with special abilities suffer from developmental disorders that are congenital in nature and cause oral health inequalities. For instance, children with Down syndrome usually suffer from open bite and dysphagia, which leads to increased plaque accumulation and calculus formation. Similarly, children with autism spectrum disorder (ASD) have poor dietary preferences, behavioral changes, regurgitation of food, bruxism, removing teeth themselves, gingival picking, etc., causing multiple oral health issues [5].

In children with intellectual disabilities, one of the most common health problems is oral disorders. The incidence of various oral diseases is higher in children with special needs than in normal children, and the increased fear of dental treatment could aggravate this condition [6].

The extent of complications and difficulties faced by children with special needs is directly proportional to the extent and type of disability. In Western countries, guidelines have been laid down for day-to-day oral healthcare for the disabled. However, there is no such provision for the prevention and treatment of various oral problems among children with special needs in India [7].

Family members as well as dentists should work together to maintain the oral health of these children, who often show damaging habits such as difficulty in movement and behavioral issues that can harm their teeth [1]. Hence, the present study will help sensitize dental professionals and caregivers of these children about the importance of dental health and the impact that poor dental health can have on their lifestyle.

Various studies have shown that children with disabilities have poor oral health [8]. The current study aims to provide additional evidence to existing studies regarding the prevalence of oral diseases among these children, while also correlating the oral health impact profile (OHIP) with the oral health status to assess the level of challenge faced by these children in maintaining their oral health. The results of this study would help fill the gap in planning treatment for such children and aid in training dentists to provide special care according to the individual challenges faced by each child.

## Materials and methods

Study design and setting

This descriptive cross-sectional study was conducted over two months at a special school in Dwarka, New Delhi, India. The study aimed to assess the oral health status and oral health-related quality of life (OHRQoL) in children with special needs.

Study population and sampling

The study population comprised children with special needs, aged less than 13 years, attending the aforementioned special school. This group was identified as a vulnerable population. A total of 135 subjects were enrolled, representing all registered children at the special school whose guardians provided informed consent for participation and who met the inclusion criteria.

Inclusion and exclusion criteria

The following are the inclusion criteria: (1) children and adolescents with special needs aged less than 13 years; (2) children with special needs attending the designated special school in Dwarka, New Delhi; and (3) children willing to participate (i.e., able to provide responses or cooperate for clinical examination, with guardian consent). The following are the exclusion criteria: (1) children unwilling to participate in the study and (2) patients with severe disabilities precluding cooperation for clinical examination.

Data collection

Data were collected through a structured questionnaire and clinical oral examination. A self-designed, structured, and interview-based questionnaire, divided into three parts, was administered. If the child was able to respond, they did so directly. Assistance from the guardian was only sought for confirmation and understanding of the response received by the subject, where required.

Part 1 (sociodemographic data) included information, such as name, age, gender, and reported level of disability. Part 2 (basic oral hygiene practices) focused on self-reported oral hygiene habits, including frequency of brushing, use of other dental aids, and reasons for past dental visits. Part 3 (the Oral Health Impact Profile - 14 items, OHIP-14) uses the OHIP-14 questionnaire [9] (©Munksgaard, 1997, as developed by Slade [3]) to assess OHRQoL. This instrument comprises 14 questions, with two questions each across seven domains: functional limitation, physical pain, psychological discomfort, physical disability, psychological disability, social disability, and handicap. Responses were recorded using a 5-point Likert scale, ranging from 0 ("never") to 4 ("very often"). The total OHIP score was calculated by summing the ordinal values of the 14 items, yielding a range from 0 to 56. Individual domain scores ranged from 0 to 8.

Questionnaire validation

As OHIP is a validated questionnaire, face validity and content validity were not performed. However, linguistic and cross-cultural adaptation was performed for the Hindi setting. Caregiver-assisted response bias was eliminated with the inclusion of only those children who were able to provide responses themselves. Reliability was evaluated using Cronbach’s alpha and a correlation matrix. The calculated Cronbach’s alpha was 0.826, and after standardization of items, it was 0.778, both exceeding the acceptable threshold of 0.7. The correlation matrix demonstrated positive correlations between most item pairs.

Reliability statistics

The reliability statistics are as follows: Cronbach's alpha = 0.826; Cronbach's alpha based on standardized items = 0.778; and N = 13.

Clinical oral examination

Clinical oral examinations were conducted by two trained examiners (one examiner and one recorder) to assess various oral health parameters. Before the study, examiners underwent training, and inter-examiner reliability was assessed using kappa statistics, ensuring appropriate agreement before data collection commenced.

Examinations were performed at the special school. Each child was accompanied by a guardian, from whom written informed consent had been obtained. Children were seated comfortably in a chair for the examination. A mouth mirror, explorer, and blunt probe were used for all clinical assessments. The following indices were utilized:

The plaque index (PI), described by Silness and Löe in 1964 [6], was employed. All teeth were assessed by running an explorer over the tooth surfaces. For each tooth, four surfaces (distobuccal, buccal, mesiobuccal, and lingual) were recorded. Plaque was scored on a scale of 0 to 3, and an average score was obtained for the entire oral cavity. Interpretation of scores was as follows: "excellent" (0), "good" (0.1-0.9), "fair" (1.0-1.9), and "poor" (2.0-3.0).

The gingival index (GI), developed by Löe and Silness in 1963 [6], was used to evaluate periodontal status. All teeth were examined by gently walking a blunt probe along the tooth surface. Similar to the plaque index, four surfaces per tooth were recorded. Scores ranged from 0 to 3, and an average score for the oral cavity was calculated. Interpretation of scores for gingivitis severity was: "mild" (0-1.0), "moderate" (1.1-2.0), and "severe" (2.1-3.0).

The decayed, missing, and filled teeth (DMFT) index, introduced by Klein, Palmer, and Knutson in 1938 [6], was calculated. Each tooth was examined with a mouth mirror and explorer and classified as decayed (D), missing (M) due to caries, or filled (F). The total score was then calculated to assess the dental caries experience of the children.

Ethical considerations and consent

The study received approval from the institutional ethics committee before its initiation. Written permission was obtained from the person in charge of the special school. Both verbal and written consent were obtained from the guardians and parents of children under 13 years of age, and the child's willingness to participate was also ensured. The entire procedure was explained to the guardians in their local language.

Statistical analysis

All collected data were analyzed using Statistical Product and Service Solutions (SPSS, version 20.0; IBM SPSS Statistics for Windows, Armonk, NY). Descriptive statistics, including percentages, means, and standard deviations, were calculated for questionnaire parameters and oral health conditions. The Shapiro-Wilk test was performed to assess data normality. As the data were found to be non-normally distributed, nonparametric tests were employed.

Spearman’s correlation coefficient was used to determine the correlation between individual OHIP domain scores, as well as the total OHIP score, and oral health outcomes (mean DMFT, plaque index, and gingival index). The Pearson chi-square test was conducted to ascertain significant differences in oral health status between those who reported an impact on OHRQoL and those who did not. Differences in OHIP scores based on gender and other sociodemographic factors were assessed using χ² analysis for proportions.

## Results

The OHIP score was calculated by summing the ordinal values of 14 items, which could range from 0 to 56. The score of individual domains ranged from 0 to 8. Once the score for each domain was calculated, the scores of all domains were added to obtain the OHIP total score [10].

A total of 135 subjects were included in the study, studying in an institutionalized special school in Dwarka. The children examined were between four and 13 years of age. Of these, 113 (84%) belonged to the age group of 10-13 years, while 22 (16%) belonged to the age group of four to nine years. Among them, 104 (77%) were male and 31 (23%) were female. Out of all the subjects examined, 119 (88%) studied in middle school and 16 (12%) in primary school (Table 1).

**Table 1:** Sociodemographic characteristics of the study population

Variables	Frequency (n)	Percentage (%)
Gender		
Male	104	77.0
Female	31	23.0
Age (in years)		
4-6 years	5	3.7
7-9 years	17	12.6
10-13 years	113	83.7
Education level		
Class 1-5	16	11.9
Class 6-10	119	88.1

About oral hygiene maintenance, 104 (77.0%) subjects reported brushing their teeth once daily, while 31 (23.0%) brushed twice daily. When asked about other dental aids, 47 (34.8%) used only a toothbrush, 12 (8.9%) used dental floss, two (1.5%) used an interdental brush, and 74 (54.8%) used mouthwash. Regarding dental visits, a significant majority of 106 (78.5%) had never visited a dentist. Only five (3.7%) had visited a dentist one month prior, one (0.7%) six months prior, and 23 (17.0%) one year prior, as shown in Table 2.

**Table 2:** Oral hygiene maintenance of the study population

Variables	Frequency (n)	Percentage (%)
Can the child do household chores on their own?		
Yes	98	72.6
No	37	27.4
How many times does the child brush their teeth?		
Once	104	77.0
Twice	31	23.0
Any other dental aid used		
Floss	12	8.9
Interdental brush	2	1.5
Mouthwash	74	54.8
Others (toothbrush)	47	34.8
When was the last visit to a dentist?		
One month ago	5	3.7
Six months ago	1	0.7
One year ago	23	17.0
Never	106	78.5

Among the 135 subjects, autism was the most prevalent disability (69, 51.1%). Other reported disabilities included learning disability (8, 5.9%), emotional disturbance (3, 2.2%), speech or language impairment (14, 10.4%), mental retardation (10, 7.4%), visual impairment (4, 3.0%), hearing disability (4, 3.0%), physical disability (5, 3.7%), and traumatic brain injury (1, 0.7%). A category of "Others" accounted for 17 (12.6%) disabilities, as detailed in Table 3.

**Table 3:** Type of disability

Type	Frequency (n)	Percentage (%)
Learning disability	8	5.9
Emotional disturbance	3	2.2
Speech or language impairment	14	10.4
Mental retardation	10	7.4
Visual impairment	4	3.0
Hearing disability	4	3.0
Physical disability	5	3.7
Autism	69	51.1
Traumatic brain injury	1	0.7
Others	17	12.6

The OHIP total score was calculated by summing the scores of its 14 items, with a possible range of 0 to 56. Individual domain scores ranged from 0 to 8. The mean OHIP total score for the study population was 5.05 ± 6.28. The mean DMFT score, calculated by summing DMFT, was 2.96 ± 2.29. The mean scores for each OHIP domain and DMFT are presented in Table 4.

**Table 4:** Mean of OHIP scores and DMFT OHIP: oral health impact profile; DMFT: decayed, missing, and filled teeth

Variable	N	Mean	Std. Deviation
Functional limitation score	135	1.10	1.554
Physical pain score	135	1.31	1.785
Psychological discomfort score	135	1.03	1.559
Physical disability score	135	0.70	1.323
Psychological disability score	135	0.30	0.650
Social disability score	135	0.34	0.848
Handicap score	135	0.27	0.706
OHIP total score	135	5.05	6.288
DMFT score	135	2.96	2.293

Correlation analysis between OHIP domains and the DMFT index revealed statistically significant positive correlations for the functional limitation score (0.048) and handicap score (0.219). For all other domains, the correlation coefficient was not statistically significant.

Based on total scores obtained for the gingival index, interpretation was done in the form of “mild gingivitis” for scores 0-1.0, “moderate gingivitis” for scores 1.1-2.0, and “severe gingivitis” for scores 2.1-3.0 [6].

On correlating the handicap score with the gingival index score, the correlation coefficient was 0.230, which was statistically significant. Based on total scores obtained for the plaque index, interpretation was as follows: “excellent” for a score of 0, “good” for a score in the range 0.1-0.9, “fair” for a score in the range 1.0-1.9, and “poor” for a score in the range 2.0-3.0 [6]. On correlating the gingival index scores with the plaque index scores, the correlation coefficient was 0.342, which was statistically significant (Table 5).

**Table 5:** Correlation of various domains of OHIP with oral health conditions **: correlation is significant at the 0.01 level (one-tailed); *: correlation is significant at the 0.05 level (one-tailed); OHIP: oral health impact profile (OHIP); DMFT: decayed, missing, and filled teeth (DMFT)

Variable	DMFT score correlation coefficient	Plaque score correlation coefficient	Gingival score correlation coefficient
Functional limitation score	0.048*	0.067	0.037
Physical pain score	0.085	0.087	0.037
Psychological discomfort score	0.005	0.107	0.005
Physical disability score	0.079	0.117	0.081
Psychological disability score	0.017	0.093	0.049
Social disability score	0.090	0.001	0.112
Handicap score	0.219**	0.073	0.230**
OHIP total score	0.031	0.045	0.040
Gingival index score		0.342**	
Plaque index score			0.342**

On correlating the gingival index with OHRQoL under the OHIP category, 56 (71.8%) subjects with mild gingivitis, 28 (66.7%) with moderate gingivitis, and all subjects with severe gingivitis reported no impact on OHRQoL (Table 6). This suggests a potential lack of awareness or perception among these children regarding the impact of their oral health conditions on their quality of life. On correlating plaque index with OHRQoL under the OHIP category, 17 (81.0%) subjects with a good plaque score, 69 (70.4%) with a fair score, and 13 (81.2%) with poor plaque scores reported no impact on OHRQoL (Table 7).

**Table 6:** Correlating the gingival index with OHRQoL OHIP: oral health impact profile; OHRQoL: oral health-related quality of life

Gingival index		OHIP category		Total
	No impact on OHRQoL		Impact on OHRQoL	
1. Normal	n	12	0	12
	%	100.00%	0.00%	100.00%
2. Mild gingivitis	n	56	22	78
	%	71.80%	28.20%	100.00%
3. Moderate gingivitis	n	28	14	42
	%	66.70%	33.30%	100.00%
4. Severe gingivitis	n	3	0	3
	%	100.00%	0.00%	100.00%
Total	n	99	36	135
	%	73.30%	26.70%	100.00%

**Table 7:** Correlating plaque index with OHRQoL OHIP: oral health impact profile; OHRQoL: oral health-related quality of life

Plaque index		OHIP category		Total
		No impact on OHRQoL	With impact on OHRQoL	
Good	n	17	4	21
	%	81.00%	19.00%	100.00%
Fair	n	69	29	98
	%	70.40%	29.60%	100.00%
Poor	n	13	3	16
	%	81.20%	18.80%	100.00%
Total	n	99	36	135
	%	73.30%	26.70%	100.00%

The findings of this study indicate a sense of satisfaction among the study subjects about their existing oral health status, without awareness of its adverse effects.

## Discussion

Special healthcare needs can be defined as a limiting condition that requires intervention in healthcare, medical management, and/or healthcare programs due to any mental, behavioral, physical, sensory, emotional, developmental, or cognitive impairment. Besides systemic diseases and condition-specific traits, oral diseases are a major issue for individuals with special needs, reflecting their mental and physical state. The current study included a study group mainly belonging to the age range of 10-13 years, with a majority of them being male. Similar study populations were seen in research conducted in Arabia by Essa et al. [1] and in Goa by Gadiyar et al. [11]. A majority of children with special needs in our study revealed that they brushed their teeth only once per day. This observation is similar to findings in other studies and shows that most of the children with special needs, such as ASD, had poorer oral health status when compared with healthy children.

In our study, the children had raised plaque scores, moderate gingivitis, and raised DMFT scores, indicating poor oral hygiene with increased incidence of caries, as well as gingival and periodontal disease problems. The increased caries incidence that we found was similar to results obtained by various studies conducted in children with intellectual disability, especially cerebral palsy and other syndromic and non-syndromic disabilities [4,12-18].

The dental professional needs to understand that various dental problems, such as dental caries, malocclusion, bruxism, and periodontal disease, can cause limitations and deficiencies among children with special demands. Although the sample size of our study was too small to validate correlations, we found that poor oral health can cause significant functional limitations and handicaps among children with special abilities by correlating DMFT scores with seven domains of OHIP. This was in contrast to the high OHIP scores seen in a cross-sectional study conducted by Eltas et al. in 2016 on 404 patients with periodontitis and gingivitis [19]. The main cause of this discrepancy could be related to the fact that children with special needs have less intellectual development and, thus, may not be able to understand the effect that poor oral hygiene has on their lives.

The reason behind our study’s raised DMFT, plaque, and gingival scores and the simultaneous high prevalence of dental caries, poor hygiene, and a high incidence of periodontal disease among these children could be due to various behavioral, physical, medical, mental, and emotional challenges faced by these children [5]. Simple actions of chewing, brushing, or brushing more than once can be an uphill task for them and their caregivers every day, similar to findings in other studies.

The results of our study also showed that children with poor oral hygiene and various gingival diseases reported no impact on OHRQoL. This was consistent with the results obtained by Cancio et al., who found that the impact on parents' quality of life was not negative when associated with a high caries experience or other socioeconomic factors [20]. It shows that children with special needs and their caregivers have an acceptance of poor oral health and are not able to understand the impact poor oral health is causing on their lifestyle.

The need of the hour is to focus on strategies to improve the oral health of these children. This highlights the need to promote awareness regarding early home oral hygiene practices among them [8]. The veracity of this was seen in a study where blind children aged 10-12 had acceptable oral hygiene due to supervised brushing [13].

It is important that caregivers and parents be sensitized by various awareness programs regarding the importance of maintaining good oral health in children with special abilities.

It is necessary and important to improve the oral health status of these children. Difficulty in communication, understanding, and a lack of interest could be areas that could prove to be major roadblocks in trying to improve the dental health of these children. This can be overcome by teaching them various preventive measures and conducting regular screening camps in special schools to enhance the quality of life of these children with special needs and sensitize them and their caregivers to the concept of good quality of life. The limitation in our study was that it was conducted at a single site catering to special needs children. Inclusion of more such centers would have allowed for more inclusive data and a better correlation of all the objectives with other disabilities seen in children with special needs.

## Conclusions

The study reveals that children with special needs have poor dental hygiene with increased risk of caries and gingival problems, irrespective of the type of disability or their ability to do their work independently. Even among autistic children who demonstrated independence in daily tasks, poor oral health was evident, suggesting a significant lack of awareness among parents, caregivers, and special institutions about the importance of oral health. Interestingly, despite their compromised oral health, most of these children reported minimal impact on their OHRQoL, indicating a potential inability to perceive or articulate the effects of poor oral health. Other reasons could be the inability of these subjects to perceive or articulate the effects of oral health. The reduced outcome of the tool to assess the quality of life in children with disability may be due to the lack of awareness about the importance of oral health among caregivers and parents themselves. Hence, inculcating adaptive behaviors in such children is compromised. Strengthening, training, and policy-making are recommended for caregivers and parents of such children for better outcomes.

Therefore, this study underscores the critical need to sensitize parents, caregivers, and dental professionals to the importance of consistent oral health monitoring and early intervention for children with special needs. Oral health is equally vital to physical and mental well-being. Targeted oral health education and awareness programs focusing on preventive measures are crucial for improving oral health outcomes in this vulnerable population. As part of this study, a small educational program was organized to demonstrate correct brushing techniques and to emphasize oral hygiene practices to both children and their caregivers.

## References

[1] Essa EZ, Al-Zuhair AM, Jaber AA, Alkhabuli J (2019). Oral health status and treatment needs for children with special needs: a cross-sectional study. Pesqui Bras Odontopediatria Clín Integr.

[2] (2025). Persons with disabilities (divyangjan) in India - a statistical profile: 2021. https://ndfdc.nic.in/upload/nhfdc/Persons_Disabilities_31mar21.pdf.

[3] Santosh A, Kakade A, Mali S, Takate V, Deshmukh B, Juneja A (2021). Oral health assessment of children with autism spectrum disorder in special schools. Int J Clin Pediatr Dent.

[4] Gaçe E, Kelmendi M, Fusha E (2014). Oral health status of children with disability living in Albania. Mater Sociomed.

[5] Ningrum V, Bakar A, Shieh TM, Shih YH (2021). The oral health inequities between special needs children and normal children in Asia: a systematic review and meta-analysis. Healthcare (Basel).

[6] Kuter B, Uzel I (2021). Evaluation of oral health status and oral disorders of children with autism spectrum disorders by gender. Arch Pediatr.

[7] Vishnu P, Mahesh R, Madan Kumar PD, Sharna N (2021). Oral health status and treatment needs of children with sensory deficits in Chennai, India-a cross-sectional study. Indian J Dent Res.

[8] Bagattoni S, Lardani L, D’Alessandro G, Piana G (2021). Oral health status of Italian children with autism spectrum disorder. Eur J Paediatr Dent.

[9] Slade GD (1997). Derivation and validation of a short-form oral health impact profile. Community Dent Oral Epidemiol.

[10] Mary AV, Mahendra J, John J, Moses J, Ebenezar AV, Kesavan R (2017). Assessing quality of life using the oral health impact profile (OHIP-14) in subjects with and without orthodontic treatment need in Chennai, Tamil Nadu, India. J Clin Diagn Res.

[11] Gadiyar A, Gaunkar R, Kamat A, Kumar A (2020). Influence of intellectual disabilities on oral health among children attending special schools in Goa: a cross-sectional study. J Indian Assoc Public Health Dent.

[12] Piraneh H, Gholami M, Sargeran K, Shamshiri AR (2022). Oral health and dental caries experience among students aged 7-15 years old with autism spectrum disorders in Tehran, Iran. BMC Pediatr.

[13] Kannappan J, Srinivasan D, Chiriyankandath JL, Arumugam SE, Natarajan D, Annamalai SS (2023). Assessment of oral hygiene status and prevalence of dental caries and traumatic injuries to anterior teeth among visually impaired children in Chennai City. Int J Clin Pediatr Dent.

[14] Makkar A, Indushekar KR, Saraf BG, Sardana D, Sheoran N (2019). A cross sectional study to evaluate the oral health status of children with intellectual disabilities in the National Capital Region of India (Delhi-NCR). J Intellect Disabil Res.

[15] Gadiyar A, Gaunkar R, Kamat AK, Tiwari A, Kumar A (2018). Impact of oral health-related behaviors on dental caries among children with special health-care needs in Goa: a cross-sectional study. J Indian Soc Pedod Prev Dent.

[16] Suresan V, Das D, Jnaneswar A, Jha K, Kumar G, Subramaniam GB (2017). Assessment of dental caries, oral hygiene status, traumatic dental injuries and provision of basic oral health care among visually impaired children of eastern Odisha. J Indian Soc Pedod Prev Dent.

[17] Solanki J, Gupta S, Arya A (2014). Dental caries and periodontal status of mentally handicapped institutilized children. J Clin Diagn Res.

[18] Nqcobo CB, Yengopal V, Rudolph MJ, Thekiso M, Joosab Z (2012). Dental caries prevalence in children attending special needs schools in Johannesburg, Gauteng Province, South Africa. SADJ.

[19] Eltas A, Uslu MO, Eltas SD (2016). Association of oral health-related quality of life with periodontal status and treatment needs. Oral Health Prev Dent.

[20] Cancio V, Faker K, Bendo CB, Paiva SM, Tostes MA (2018). Individuals with special needs and their families’ oral health-related quality of life. Braz Oral Res.

